# Effects of encapsulated *Lavandula angustifolia* essential oil in alginate hydrogel capsules as feed additives to enhance the performance and health of broiler chickens and its *in vitro* antibacterial activity against multidrug-resistant *Escherichia coli* strains

**DOI:** 10.1016/j.psj.2026.106510

**Published:** 2026-01-24

**Authors:** Michalina Adaszyńska-Skwirzyńska, Sławomir Zych, Marta Grabowska, Mateusz Bucław, Danuta Majewska, Danuta Szczerbińska, Adam Lepczyński, Paweł Konieczka

**Affiliations:** a Department of Monogastric Animal Sciences, Faculty of Biotechnology and Animal Sciences, West Pomeranian University of Technology in Szczecin, Janickiego St. 29, 71-270, Szczecin, Poland; b Department of Microbiology and Biotechnology, Faculty of Biotechnology and Animal Sciences, West Pomeranian University of Technology in Szczecin, Piastów Ave. 45, 70-310, Szczecin, Poland; c Department of Histology and Developmental Biology, Pomeranian Medical University, Żołnierska St. 48, 71-210, Szczecin, Poland; d Department of Physiology, Cytobiology and Proteomics, Faculty of Biotechnology and Animal Sciences, West Pomeranian University of Technology in Szczecin, Janickiego St. 29, 71-270, Szczecin, Poland; e Department of Poultry Science and Apiculture, University of Warmia and Mazury in Olsztyn, Oczapowskiego St. 5, 10-719, Olsztyn, Poland; f The Kielanowski Institute of Animal Physiology and Nutrition, Polish Academy of Sciences, Instytucka St. 3, 05-110, Jabłonna, Poland

**Keywords:** Broiler chicken, Encapsulation, *Escherichia coli*, Lavender (*Lavandula angustifolia* L.) essential oil, multidrug-resistant

## Abstract

The study aimed to evaluate the *in vivo* effects of the addition of lavender essential oil (**LEO**) immobilized in alginate hydrogel administered during the first period of rearing on production performance, selected blood parameters, gut microflora, and jejunum morphology in broiler chickens, as well as to assess its *in vitro* antibacterial activity against clinical *Escherichia coli* strains with varying drug resistance isolated from 1-day-old chicks. The experiment was conducted on a commercial farm using 300 unsexed Ross 308 broilers. One-day-old chicks were assigned to three experimental groups of 100 birds each, with five replicates of 20 birds per group. In the control group (**CON**), the chicks received feed without essential oil supplementation throughout the rearing period. In the experimental groups hydrogel (**H**) and hydrogel with LEO (**HE**), 2 % (w/w; relative to the other feed components) of alginate hydrogel capsules were added to the starter feed mixture (days 1–10). During the experiment, body weight (**BW**), feed intake (**FI**), water intake (**WI**), and mortality of the chicks were recorded. At the end of the starter feed period (day 10), blood and jejunum samples were collected from the chicks for analysis of selected biochemical, microbiological, and morphological parameters.

The results demonstrated that supplementation with hydrogel containing immobilized LEO positively affected the feed conversion ratio (**FCR)** (*P* < 0.05), while no differences were observed between the groups in FI, mortality, or blood biochemical parameters (*P* > 0.05). It was shown that supplementation with hydrogel capsules containing immobilized LEO reduced the counts of *E. coli* and coliforms in intestinal samples (*P* < 0.05). No differences were observed in the counts of lactic acid bacteria (*P* > 0.05), and no relevant morphological changes were detected in the liver or jejunum. LEO was effective in inhibiting the growth of all *E. coli* strains, with minimal inhibitory concentration (**MIC**) values ranging from 1.0 to 4.0 % (v/v).

## Introduction

New lines of broiler chickens are characterized by increased growth potential but are also more susceptible to various disorders and diseases, particularly those affecting the digestive system, such as colibacillosis (Fancher et al., 2020). Gut health encompasses a range of physiological and functional traits that directly influence chicken growth rate, particularly in intensive poultry production systems. The small intestine is the primary site for regulating digestion and nutrient absorption in poultry and tightly controls the transport of proinflammatory molecules, microorganisms, and toxins, functioning as a barrier against pathogens (Glendinning et al., 2019; Shang et al., 2018). *Escherichia coli* is part of the normal gut microbiota in poultry, and most strains are non-pathogenic. However, certain serovars can cause disease in broiler chickens, whereby *E. coli* may induce yolk sacculitis, coligranulomatosis, salpingitis-peritonitis, or colisepticemia (Lutful Kabir, 2010). All these conditions can be grouped under the term colibacillosis. Bacterial infections, particularly colibacillosis, are among the main disease factors affecting gut health and impairing the production performance and immune system of poultry (Kromann and Jensen, 2022). In intensive production systems, broilers are exposed to stress factors such as feed changes, transportation, and stocking density. These factors can negatively affect both humoral and cellular immune responses, making the broiler gut susceptible to colonization by bacterial pathogens such as *Salmonella* spp., *E. coli, Campylobacter jejuni*, and *Clostridium perfringens*. One of the most significant challenges in modern poultry production is colibacillosis, which ranks among the most frequently diagnosed disease entities in broiler chickens (Adaszyńska-Skwirzyńska et al., 2021).

Consequently, as modern poultry farming methods aim to reduce the use of chemical agents in animal nutrition, interest has grown in recent years in natural plant-based products that can be used as phytobiotic feed additives. Among these additives, herbs are of particular relevance, as they are increasingly used in feed mixtures and supplements due to their content of valuable bioactive compounds, such as essential oils. Essential oils (**EOs**) show diverse biological activity (Adaszyńska-Skwirzyńska and Szczerbińska, 2019; Adaszyńska-Skwirzyńska et al., 2021; Salinas-Chavira et al., 2024). Their use in animal nutrition can improve feed taste and aroma, increase intake, regulate digestive functions, modulate gut microbiota, and consequently influence growth performance and feed efficiency, which is especially important given rising feed costs (Salinas-Chavira et al., 2024; Adaszyńska-Skwirzyńska and Szczerbińska, 2019). EOs can show antibacterial activity and provide protection against the harmful effects of pathogenic microorganisms (Obianwuna et al., 2024). Lavender essential oil (**LEO**) extracted from *Lavandula angustifolia* flowers is among the popular biologically active EOs, representing the most widespread secondary metabolite product of this plant (Carrasco et al., 2016). Narrow-leaved lavender, *Lavandula angustifolia* Mill. (formerly *Lavandula officinalis* Chaix or *Lavandula vera*), is commonly known as true, medicinal, or common lavender. It is currently cultivated in various regions worldwide, including the United States, Australia, France, Bulgaria, Japan, and almost all areas of Poland. LEO is a volatile bioactive substance with a characteristic aroma (Prusionowska and Śmigielski, 2014; Miastkowska et al., 2021). Chemically, it is a complex mixture of terpenoid compounds, including monoterpenes, sesquiterpenes, and their oxygenated derivatives, classified according to isoprenoid units (Adaszyńska-Skwirzyńska et al., 2019). The compounds in LEO include hydrocarbons (e.g., myrcene, α-pinene, caryophyllene), alcohols (e.g., linalool, α-terpineol, borneol), ketones (e.g., camphor, carvone, eucalyptone), esters (e.g., linalyl acetate, lavandulyl acetate, geranyl acetate), aldehydes (e.g., neral), oxides (e.g., caryophyllene oxide), and ethers (e.g., eucalyptol). In addition to the compounds listed, coumarins and organic acids are also present (Adaszyńśka-Skwirzyńśka and Szczerbińska, 2017). Literature indicates that LEO has antibacterial, antifungal, antioxidant, anti-inflammatory, and spasmolytic properties (Prusinowska and Śmigielski, 2014; Carrasco et al., 2016; Adaszyńska-Skwirzyńska and Szczebińska, 2019; Miastkowska et al., 2021). Studies in animals and humans indicate that lavender exerts potential immunostimulatory, anxiolytic, calming, sedative, analgesic, anticonvulsant effects and improves mood (Carrasco et al., 2016; Adaszyńska-Skwirzyńska et al., 2024; Obianwuana et al., 2024). LEO is a volatile and lipophilic biologically active substance, which limits its practical application. However, encapsulation technologies, such as hydrogels, can be used to preserve its properties.

Hydrogels are biopolymers that can be defined as water-swollen polymeric networks produced through a simple monomer polymerization (Ahmed, 2015). An alternative definition describes hydrogel as a polymeric material characterized by its ability to swell and retain significant amounts of water within its three-dimensional network without dissolving in it. Hydrogels are also classified as colloidal systems in which water acts as the dispersion medium (Savić Gajić et al., 2023). Hydrogels based on chitosan, collagen, or alginates show significant application potential. Alginates are naturally occurring polysaccharides. Sodium alginate is the salt of alginic acid, naturally found in the cell walls of marine brown algae (*Phaeophyceae*) and in the mucilage of certain bacterial species. Alginate hydrogels can be produced through ionic or covalent crosslinking (Adaszyńska-Skwirzyńska et al., 2025a). In the former, sodium alginate exchanges its sodium ions for calcium ions in a reaction occurring when an aqueous solution of sodium alginate is mixed with a calcium chloride solution. Alginate hydrogels are increasingly used as delivery systems for bioactive compounds due to their biocompatibility, biodegradability, and capacity to protect volatile substances from oxidation and rapid degradation. In animal nutrition, alginate-based encapsulation can improve the stability and intestinal availability of EOs, enabling their gradual release in the gastrointestinal tract (Adaszyńska-Skwirzyńska et al., 2025a). In recent years, hydrogels have increasingly become a subject of interest for scientists and technologists (Ahmed, 2015; Hesabi et al., 2022; Savić et al., 2023; Adaszyńska-Skwirzyńska et al., 2025a; Adaszyńska-Skwirzyńska et al., 2025b; Cao et al., 2025; Marzok et al., 2025). However, the literature contains only a few studies on the use of hydrogels in poultry production (Ibrahim et al., 2021; Hesabi et al., 2022; Özlü et al., 2022; Adaszyńska-Skwirzyńska et al., 2025a; Adaszyńska-Skwirzyńska et al., 2025b; Marzok et al., 2025). For instance, Özlü et al. (2022) observed that hydrogel supplementation was ineffective in chicks. Ibrahim et al. (2021) reported that supplementation with alginate hydrogel containing garlic at 400 mg/kg significantly mitigated the negative effects of necrotic enteritis caused by *C. perfringens*, resulting in improved bird growth performance. Feed additives are an integral part of the nutrition of livestock in every modern farm. Their use is associated with numerous benefits, supporting animal welfare and the improvement of economic efficiency. However, selecting the appropriate additive and its concentration is often challenging, as it requires extensive research to confirm its activity, not only *in vitro* but most importantly, in practical farming environments (*in vivo*). Therefore, the study aimed to evaluate the *in vivo* effects of LEO immobilized in alginate hydrogel, administered during the first period of rearing, on production performance, selected blood parameters, gut microbiota, and jejunum morphology in broiler chickens, and to evaluate its *in vitro* antibacterial activity against clinical *E. coli* strains with different drug resistance isolated from 1-day-old broiler chickens.

## Materials and methods

### Essential oil and its immobilization in hydrogel

A commercial LEO (Avicenna Oil, Wrocław, Poland) was used in the study. According to the manufacturer, the oil was extracted from the flowers using steam distillation. Additionally, the antibacterial activity tests included two main components of the oil, namely linalool (purity 99.9 %) and linalyl acetate (≥99 %) (Sigma-Aldrich, Saint Louis, MO, USA). The chemical composition of the LEO was determined using gas chromatography–mass spectrometry (GC-MS) using conditions described in a previous publication (Adaszyńska-Skwirzyńska et al., 2025a). Data collection and processing were performed using the ChemStation software. Compounds in the samples were identified based on their mass spectra, which were compared with reference spectra from the NIST 04 library, and by using calculated retention indices. To confirm compound identification, retention indices were calculated and compared with literature data (Babushok et al., 2011). The retention times of the respective n-alkanes were determined by analyzing an n-alkane standard C7–C30 (Supelco, Bellefonte, USA) under the same chromatographic conditions. The relative percentage content of the compounds was determined based on the ratio of their peak areas to the total ion current of all compounds present in the sample. LEO was immobilized in an alginate hydrogel at 0.4 mL/L of water using Tween 80 (Sigma-Aldrich, St. Louis, USA) as an emulsifier, applied in a 1:1 ratio with LEO. The capsules were produced by APRS S.A. (Nielbark, Poland) in collaboration with the West Pomeranian University of Technology in Szczecin. The resulting capsules had a diameter of 0.25 cm.

### Antibacterial activity of essential oil and its main chemical constituents

The antibacterial activity of LEO and its two main chemical constituents was tested against 19 field strains of Gram-negative *E. coli*, isolated in 2021–2022 from clinical cases of omphalitis and yolk sac infection in one-day-old broiler chicks at a veterinary diagnostic laboratory (Września, Poland). The final strain tested was the reference strain *E. coli* ATCC 25922 (WDCM 00013; KWIK-STIK™ Plus, Microbiologics, St. Cloud, Minnesota, USA). Antimicrobial resistance data were obtained from official test reports: strains numbered 1–10 were classified as multidrug-resistant, while strains 11–19 and the reference strain (20) *E. coli* ATCC 25922 were sensitive to antimicrobial agents used in poultry treatment (Table 1). The susceptibility of the bacterial strains to the recommended antibiotics and chemotherapeutics was determined using the disk diffusion method and verified according to CLSI guidelines (Clinical and Laboratory Standards Institute (CLSI) 2018, Clinical and Laboratory Standards Institute (CLSI) 2021). The method for determining antibacterial activity was described in detail in a previous publication (Adaszyńska-Skwirzyńska et al., 2025d). Isolates were preserved using ViaBank™ bacterial strain archiving kits (MVE, Medical Wire & Equipment, Potley Lane, Corsham, England) and stored at temperatures below -20°C until LEO activity analysis. Twenty-four hours before inoculating the test plates, each strain was sequentially revived on Columbia agar supplemented with 5 % sheep blood (Graso, Starogard Gdański, Poland) and incubated at 37°C ± 1°C. The LEO and reference compounds were initially diluted in acetonitrile (LiChrosolv®, Supelco, Merck KGaH, Darmstadt, Germany) to a working concentration of 40 % v/v. The antibacterial activity of LEO, reference standards, and pure controls was determined using the minimum inhibitory concentration (**MIC**) method on sterile 96-well plates with lids (Wuxi Nest Biotechnology, Wuxi, China). Mueller-Hinton broth (**MHB**) (GRASO, Gdansk, Poland) was used as the culture medium. In the first row of wells, 180 µL of MHB and 20 µL of each working dilution of the essential oil or reference standards were added individually, resulting in an initial concentration of 4 % v/v of the test substance in the horizontal layout of the plate. The content was thoroughly mixed using a multichannel pipette, and 100 µL of the resulting solution was transferred to the next row containing 100 µL of MHB and mixed well. This procedure was repeated for successive rows until a dilution gradient from 4 % to 0.06 % (v/v) was achieved across the plate. The last two rows served as positive and negative controls, with all wells containing only 100 µL of MHB without any antimicrobial agent. However, to exclude the negative influence of acetonitrile on bacterial growth, the positive control wells contained an addition of 5 % acetonitrile. The prepared test plate was inoculated by adding 10 µL of the selected *E. coli* strain, previously standardized to 0.5 McFarland and then diluted tenfold to obtain the working bacterial suspension. As a result, the final bacterial concentration in each well was approximately 1.0 × 10^6^ cfu/mL. Bacteria were not added to the last row of the plate, serving as a negative control to verify the medium sterility and the environmental conditions of the assay. The plates were incubated for 18 h at 36°C ± 1°C. After incubation, 20 µL of sterile 0.01 % resazurin solution (POL-AURA, Olsztyn, Poland) was added to each well, and the plates were incubated for an additional 6 h. Resazurin is a dark blue dye that changes to pink only in the presence of viable cells. The MIC was defined as the lowest dilution of the EO at which the color of the bacterial culture remained unchanged (blue) after a total of 24 hours of incubation. Each MIC assay was performed in triplicate.

**Table 1:** Drug susceptibility of selected isolates of *Escherichia coli* (n=19) and the ATCC 25922 reference strain.

^1^Antimicrobial agent: AMX – amoxicillin, AMC – amoxicillin/clavulanic acid, ENR – enrofloxacin, SXT – sulfamethoxazole/trimethoprim, DO – doxycycline, OT – oxytetracycline, FFC – florfenicol, CT – colistin, LS – lincomycin/spectinomycin.

### Study design

The experiment was conducted on a commercial farm (Veterinary Identification Number 32044946) under the supervision of the district veterinarian in Goleniów and the West Pomeranian University of Technology in Szczecin. Approval was obtained from the Local Ethics Committee for Animal Experiments in Poznań (protocol number PL12/10/2023). The study complied with ethical standards, ensuring that the animals experienced no pain, suffering, distress, or lasting harm. Feed and water were provided ad libitum. All animal procedures were carried out in full compliance with Act No. 1580/2023 on the protection of animals against cruelty. The study was conducted in accordance with the ARRIVE 2.0 guidelines. The experiment was conducted over a standard production cycle using 300 non-sexed Ross 308 broiler chicks. Unsexed chicks were used to reflect standard commercial broiler production conditions, where flocks are commonly reared as mixed-sex groups. To minimize potential sex-related bias, chicks were randomly allocated to experimental groups, ensuring similar initial body weights. The birds were obtained from a commercial hatchery (Park Drobiarski Sp. z o.o., Śmiłowo, Poland). Throughout the experiment, the birds were under constant supervision by the District Veterinary Officer in Goleniów for clinical observation and health assessment. The chicks were kept in the production hall in designated pens. One-day-old chicks were randomly assigned to three experimental groups of 100 birds each, with five replicates of 20 birds per group. In the control group (CON), broilers did not receive any hydrogel supplementation throughout the rearing period. In groups H and HE, 2 % (w/w) hydrogel capsules were added to the feed from day 1 to day 10 of life (the feed was mixed daily with the capsules). Group H received alginate hydrogel capsules without immobilized EO, whereas group HE received hydrogel capsules containing immobilized LEO. On the last day of starter feed administration (day 10), blood was collected from the wing vein of five birds per subgroup, and the jejunum and liver were dissected following euthanasia by decapitation. The chicks had continuous access to drinking water during the entire experiment. The birds were kept in the same facility for 40 days on wheat straw bedding, at a stocking density of 14 birds/m^2^. Broilers were maintained under standardized environmental conditions in accordance with the Ross 308 rearing guidelines (Aviagen 2019). The chicks were fed ad libitum with diets from a commercial feed producer (Polskie Zakłady Zbożowe Sp. z o.o., Wałcz, Poland): starter (days 1–10), grower I (days 11–20), grower II (days 21–30), and finisher (days 31–40). The ingredient composition and nutritional value of the diets are provided in Table 2. During the experiment, broiler body weight (on days 1, 7, 14, 21, 28, 35, and 40), feed intake (FI), water consumption (WI), and mortality were recorded. Based on the data collected for the entire production cycle, feed conversion ratio (FCR) (kg/kg), mortality rate (%), survival rate (%), and European Production Efficiency Factor (EPEF) were determined. FCR was calculated as the total amount of feed consumed per kilogram of body weight gain (BWG). EPEF is an indicator of the production performance of a flock (Adaszyńska-Skwirzyńska et al., 2025c). The index was calculated using the following formula: EPEF = (average BW (kg) × survival rate (%)) / (days of rearing × FCR) × 100.

**Table 2:** Ingredient and nutrient compositions of the basal diets (%).

Item	Starter	Grower I	Grower II	Finisher
	(0–10 d)	(11–20 d)	(21–32 d)	(31–40 d)
Ingredient				
Wheat, 11.6 % CP	37.00	38.50	34.50	41.50
Soybean meal, 46 % CP	29.05	24.94	27.16	24.0
Corn, 7.8 % CP	25.93	27.39	28.74	25.32
Vegetable oil	2.68	2.78	3.79	3.66
Canola meal, 32.5 % CP	-	2.50	2.50	3.01
Potato protein, 73 % CP	1.50	1.0	0.50	-
Limestone	1.27	0.85	0.78	0.67
Monocalcium phosphate	0.92	0.43	0.30	0.11
Vitamin and mineral premix^1^	0.53	0.53	0.60	0.59
L-Lys-HCl	0.39	0.40	0.41	0.43
DL-Met	0.26	0.17	0.18	0.17
Salt	0.24	0.27	0.28	0.28
NaHCO_3_	0.14	0.13	0.15	0.14
Thr	0.07	0.06	0.07	0.07
Choline chloride	-	0.03	0.02	0.03
Phytase premix^2^	0.02	0.02	0.02	0.02
Total	100	100	100	100
Calculated analysis				
ME (kcal/kg)	3000.0	3100.0	3150.1	3200.0
Lys	1.40	1.26	1.17	1.10
Met	0.54	0.50	0.47	0.45
Ca	0.96	0.84	0.78	0.72
P	0.48	0.42	0.39	0.36
Na	0.16	0.16	0.16	0.16
Chemical analysis^3^				
Crude protein	21.96	19.80	19.00	18.05
Crude fiber	2.90	3.00	3.41	3.44
Crude fat	4.84	5.60	7.18	7.36
Crude ash	5.51	4.87	4.88	4.30

1 Vitamin-mineral premix contained the following per kilogram of diet: vitamin A, 13,000 IU; vitamin D3, 5,000 IU; vitamin E, 65 mg; vitamin B1, 3 mg; vitamin B2, 8.6 mg; vitamin B6, 3 mg; vitamin B12, 17 μg; nicotinic acid, 60 mg; pantothenic acid, 14.7 mg; folic acid, 1.5 mg; iron, 63 mg; copper, 15 mg; cobalt, 1.0 mg; zinc, 100 mg; iodine, 1.0 mg; selenium, 0.3 mg, antioxidant (BHA).

2 Phytase premix was prepared by dilution with calcium carbonate to contain 1,000 FTU (phytase units)/g (Optiphos, Huvepharma AD, Sofia, Bulgaria).

3 Based on a DM content of 87.5 %.

### Blood biochemistry and enumeration of bacteria

At the end of the starter feed period (day 10), blood and jejunum samples were collected from the chicks for analysis of selected biochemical, microbiological, and morphological parameters. Blood biochemical parameters were measured using the VetTest 8008 (Idexx Laboratories, Inc., West- brook, USA) chemistry analyzer with dryslide technology using the Idexx Laboratories methodology. Jejunum samples were collected for microbiological analyses aimed at preselected groups of microorganisms (*E. coli*, coliform, and lactic acid bacteria). Methodology was described in a previous publication (Adaszyńska-Skwirzyńska and Szczerbińska, 2019).

### Morphological analysis

The jejunum and liver were fixed in 4 % buffered paraformaldehyde and embedded in paraffin blocks. Sections (3 µm thick) were cut from the blocks for further analysis. Jejunum and liver sections were deparaffinized in xylene and then dehydrated through a series of decreasing ethanol concentrations. Subsequently, sections were stained using standard protocols: hematoxylin and eosin (**H&E**) for general tissue morphology assessment, periodic acid–Schiff (**PAS**) for polysaccharides and glycoproteins, and Masson’s trichrome to visualize collagen fibers. Sections stained with H&E and Masson’s trichrome were scanned at 400 × magnification (0.25 μm/pixel resolution) using a ScanScope AT2 scanner (Leica Microsystems, Wetzlar, Germany). The resulting digital images were analyzed on a monitor using ImageScope software (Aperio Technologies, Vista, CA, USA). The intestinal diameter and muscular layer thickness (µm) were measured on PAS-stained tissue sections using the “ruler” tool. A total of 30 measurements of muscle thickness at various locations and 20 measurements of diameter were performed in each of the studied groups. For the quantitative assessment of collagen content in the intestines and liver stained with Masson’s trichrome method, the positive pixel count v9 algorithm (Aperio Technologies, Vista, CA, USA) was used. The analysis parameters were adjusted to match visual assessment, and measurement areas were selected manually. The percentage of collagen-positive areas based on Masson’s trichrome staining (blue-stained fibers) was calculated in 20 randomly selected fields of view for each group.

### Statistical analysis

Statistical analyses were performed using PQStat software, version 1.8.4.152. Nonparametric methods were applied due to deviations from normality and unequal variances between the groups. The results were compared between the groups using the Kruskal-Wallis test followed by Dunn’s post-hoc test with Bonferroni correction; additionally, the Conover rank variance test was used. Daily feed and water intake between groups were analyzed using the Friedman test followed by Dunn’s post-hoc test with Bonferroni correction, and absolute agreement and consistency were additionally assessed using the intraclass correlation method. A probability level of *P* < 0.05 was considered statistically significant.

## Results

### Chemical composition of essential oil and antimicrobial activity

The chemical composition of LEO is presented in Table 3. Chromatographic analysis showed that the main chemical components of *L. angustifolia* flower EO were linalyl acetate (17.8–31.0 %) and linalool (28.5–33.0 %). Compounds present at lower concentrations (<5 %) included lavandulyl acetate, borneol, and caryophyllene oxide. The sensitivity of *E. coli* strains to the LEO used for immobilization in the hydrogel is presented in Table 4. LEO and its main chemical constituents showed varying antibacterial activity. Both the reference strain (ATCC 25922) and all clinical isolates (1–19) were susceptible to the substances tested. LEO was effective in inhibiting the growth of all tested strains (MIC 1.0–4.0 % v/v). All multidrug-resistant strains had MIC values for the essential oil ranging from 1.0 % to 4.0 % (v/v), whereas isolates susceptible to antimicrobials showed MIC between 1.0 % and 2.0 % (v/v). Among the main components, linalool was the most active compound, with MIC ranging from 0.125 % to 0.5 % (v/v) for both multidrug-resistant and antibiotic-susceptible strains.

**Table 3:** Retention parameters of compounds identified in lavender essential oil (*Lavandula angustifolia*) obtained by GC-MS method.

Compound	Retention index – RI^1^	Reference retention index - RI Ref 2
α-Pinene	934	935
Camphene	946	950
β-Pinene	976	977
β-Myrcene	989	989
Hexyl acetate	1008	1010
δ-3-Carene	1010	1011
*p*-Cymene	1022	1024
Limonene	1028	1029
Eucalyptol	1031	1031
Trans-β-Ocimene	1037	1038
Cis-β-Ocimene	1047	1048
Linalool oxide	1074	1075
Linalool	1101	1099
Camphor	1145	1144
Borneol	1165	1166
4-Terpineol	1177	1177
*p*-Cymen-8-ol	1183	1184
α -Terpineol	1191	1191
Linalyl acetate	1256	1256
Bornyl acetate	1280	1282
Lavandulyl acetate	1289	1291
Cuminic alcohol	1293	1295
Nerol acetate	1362	1364
Geraniol acetate	1379	1380
α-Copaene	1381	1381
β-Caryophyllene	1419	1420
(E)-β-Farnesene	1455	1456
Caryophyllene oxide	1577	1577
Epi-bicyclosesquiphellandrene	1641	1642

1 Linear retention index determined experimentally in relation to n-alkanes (C_7_-C_30_) on a HP5-MSI column.

2 Reference linear retention index from the literature: Babushok et al. (2011).

**Table 4:** Susceptibility of *Escherichia coli* strains isolated from poultry to lavender essential oils (LEO) and their main constituents, as determined by the minimum inhibitory concentration (MIC).

*Escherichia coli* strain		MIC^1^ (% v/v)		
		Lavender essential oil (LEO)	Linalool	Linalyl acetate
*Multidrug-resistant strains*				
1		2	0.5	2
2		2	0.25	1
3		2	0.25	4.0
4		2	0.25	1.0
5		1	0.25	0.50
6		1	0.25	1.0
7		1	0.125	1.0
8		4	0.25	4.0
9		2	0.50	4.0
10		2	0.25	4.0
*Susceptible strains*				
11		1	0.25	0.50
12		1	0.125	0.50
13		2	0.50	1.0
14		1	0.25	1.0
15		2	0.25	1.0
16		1	0.25	1.0
17		2	0.50	1.0
18		1	0.25	1.0
19		2	0.50	1.0
ATCC 25922		2	0.50	2.0
Summary				
Mean MIC^1^		1.57	0.29	1.27
SD^2^		0.73	0.13	1.28
CV^3^ %		46.5	44.8	100
Most frequent MICs	% v/v	2.0	0.25	1.0
	% of cases	55	60	55

1 MIC – minimal inhibitory concentration.

2 SD – standard deviation (*n* = 3).

3 CV – coefficient of variation.

### Performance parameters

The production performance results are presented in Table 5. Chick weights were uniform on the first day of rearing, averaging 43.10 g. After 10 days, chicks supplemented with hydrogel capsules reached significantly higher body weights (*P* < 0.05). At the end of the starter feeding period (first stage of rearing), chick body weights ranged from 233.8 g in the control group (CON) to 237.33 g in group H. Chicks fed the diet supplemented with hydrogel containing immobilized LEO achieved the highest body weight at the end of the rearing period, 2971.33 g, while group H reached 2891.03 g. No significant differences were observed between groups in total feed intake during the entire rearing period (*P* > 0.05). The inclusion of feed supplemented with immobilized essential oil significantly improved FCR, reducing it to 1.53 kg/kg compared with 1.58 kg/kg in the CON and H groups. Furthermore, supplementation with hydrogel capsules significantly reduced water intake during the first stage of rearing in groups H and HE, while no significant differences were observed over the entire rearing period (*P* > 0.05). Supplementation significantly affected the EPEF, which ranged from 447.46 to 475.75. Average chick mortality did not differ between groups, remaining at 2 % across all experimental treatments.

**Table 5:** Effect of alginate hydrogel supplementation on broiler growth.

Measurement per period (day)		Group^1^		SEM^2^	*P-*value^3^		
	CON	H	HE		CON vs. H	H vs. HE	HE vs. CON
Body Weight (g/bird)							
1	43.17	43.08	43.05	0.33	1.0000	1.0000	1.0000
10	233.83	237.33	237.05	0.92	0.1272	1.0000	0.2137
40	2887.1	2891.03	2971.33	19.11	0.8932	0.0497	0.0461
Feed Intake (g/day per bird)							
1-10	27.74	26.43	26.30	4.84	1.0000	1.0000	0.7158
1-40	107.07	107.12	107.04	0.66	1.0000	1.0000	1.0000
Feed Conversion Ratio (g/g)							
1-10	1.0677	0.9925	0.9986	0.004	0.2989	1.0000	0.2998
1-40	1.58	1.58	1.53	0.03	1.000	0.0434	0.0434
Water Intake (ml/day per bird)							
1-10	70.37	67.17	66.98	7.78	0.0454	1.0000	0.0434
1-40	261.6	263.32	262.71	16.51	1.0000	1.0000	1.0000
EPEF scores							
1-40	447.67	448.29	475.75	0.95	0.9981	0.0466	0.0421

1 CON: Control; H: Hydrogel; HE: Hydrogel + LEO.

2 SEM – standard error of the mean (*n* = 10).

3 Results are means of 5 replicates per treatment.

### Blood biochemistry and enumeration of bacteria

Table 6 presents the blood biochemical parameters of chickens after 10 days of the experiment. The concentrations of individual parameters were as follows: alanine transaminase (U/L) ranged from 14.13 in group HE to 17.25 in group CON; aspartate aminotransferase (U/L) from 213.62 in group HE to 223.87 in group CON; creatinine (mg/dL) from 0.41 in group CON to 0.46 in group HE; and uric acid (mg/dL) from 4.81 in group HE to 5.54 in group CON. Statistical analysis showed no significant differences in blood biochemical parameters, indicating normal liver and kidney function.

**Table 6:** Biochemical blood parameters of broiler chickens at 10 days of age.

Blood serum parameters		Group^1^		SEM^2^	*P*-value^3^		
	CON	H	HE		CON vs. H	H vs. HE	HE vs. CON
Alanine transaminase (U/L)	17.25	15.12	14.13	1.46	0.1265	1.0000	0.1681
Aspartate aminotransferase (U/L)	223.87	217.5	213.62	6.29	1.1677	0.3716	1.5393
Creatinine (mg/dl)	0.41	0.44	0.46	0.02	0.9168	1.0000	0.7135
Uric acid (mg/dl)	5.54	5.02	4.81	0.62	1.0000	1.0000	1.0000

1 CON: Control; H: Hydrogel; HE: Hydrogel + LEO.

2 SEM – standard error of the mean (*n* = 10).

3 Results are means of 5 replicates per treatment.

The results of the quantitative microbiological analysis of the jejunal microbiota in 10-day-old chickens are presented in Table 7. Microbial counts (CFU/g) in the jejunum of chicks were as follows: *E. coli* ranged from 5.95 in group HE to 6.64 in CON, coliforms from 1.44 in group HE to 1.59 in CON, and probiotic bacteria from 8.13 in group H to 8.14 in groups HE and CON. The total counts of probiotic bacteria in the groups receiving hydrogel capsules were comparable to the control group. Statistically significant differences were observed only for *E. coli* and coliforms counts in group HE compared with groups H and CON (P < 0.05).

**Table 7:** Bacterial quantification (CFU/g) in ileum content at d 10.

Microorganisms (CFU/g)	Group^1^			SEM^2^	*P*-vaule^3^		
	CON	H	HE		CON vs. H	H vs. HE	HE vs. CON
*Escherichia coli*	6.64	6.52	5.95	0.03	1.0000	0.0088	0.0004
Coliform	1.59	1.56	1.44	0.05	0.954	0.0388	0.0021
*Lactobacillus* spp.	8.14	8.13	8.14	0.02	0.8589	1.0000	1.0000

1 CON: Control; H: Hydrogel; HE: Hydrogel + LEO.

2 SEM – standard error of the mean (*n* = 10).

3 Results are means of 5 replicates per treatment.

### Morphological analysis

**Fig. 1:**
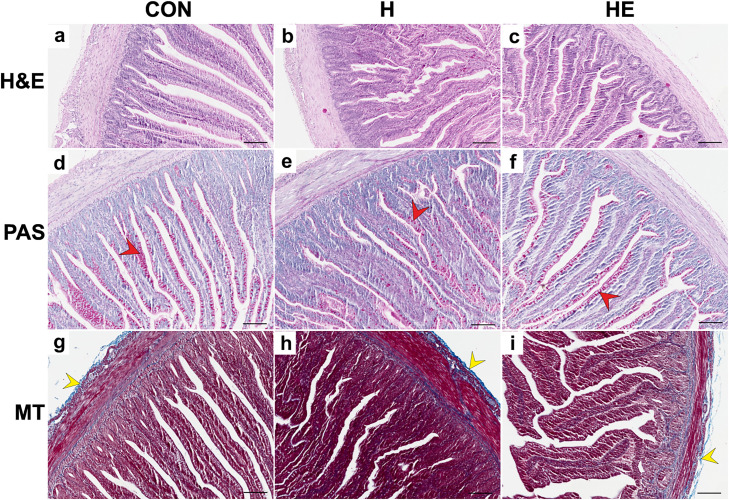
Representative microphotographs showing cross-sections of the jejunum in chicks from the control group (CON; a, d, g), supplemented with hydrogel (H; b, e, h), and hydrogel containing immobilized lavender essential oil (HE; c, f, i). Sections were stained with hematoxylin and eosin (H&E; a–c), PAS (d–f), and Masson’s trichrome (MT; g–i). In all groups, goblet cells in intestinal sections stained using the PAS method appeared dark pink (red arrowhead). In the intestinal sections stained with Masson’s trichrome method, collagen fibers were stained blue (yellow arrowhead). Scale bar – 50µm.

**Fig. 2:**
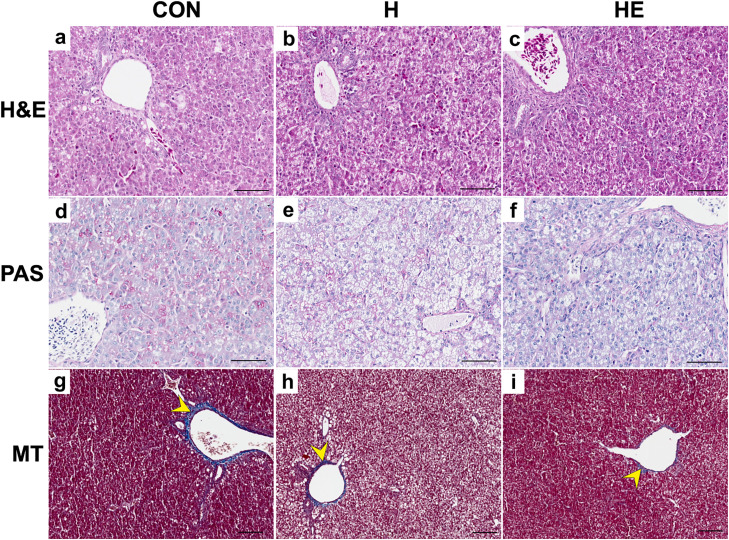
Representative microphotographs showing liver cross-sections of broilers from the control group – CON (a, d, g), supplemented with hydrogel – H (b, e, h), and hydrogel containing immobilized lavender essential oil – HE (c, f, i). Staining methods: haematoxylin and eosin – H&E (a-c), PAS method (d-f), and Masson’s trichrome – MT (g-i). In all groups, collagen fibers in liver sections stained with Masson’s trichrome appeared blue (yellow arrowhead). Scale bar – 50µm.

Morphological analysis of the intestines and liver from 10-day-old broilers (Table 8) showed that, regardless of feed supplementation, no changes were observed in the thickness of the muscularis, intestinal villi length, crypt depth, or collagen content. Hydrogel supplementation was observed to influence the width of jejunal villi in chickens. The widest intestinal villi, compared to group CON, were found in the intestines of chickens fed diets supplemented with hydrogel capsules both without EO (group H) and with immobilized LEO (group HE) (111.47 μm and 109.16 μm, respectively). No significant morphological changes were observed in either the liver or the jejunum (Fig. 1, Fig. 2).

**Table 8:** Results of the morphological analysis of organs collected from 10-day-old broilers.

Item		Group^1^		SEM^2^	*P*-value^3^		
	CON	H	HE		CON vs. H	H vs. HE	HE vs. CON
Intestinal muscularis thickness[μm**]**	114.33	116.45	114.75	3.22	1.0	1.0	1.0
Intestinal diameter [μm]	3748.9	3741.2	3842.95	92.62	1.0	0.8746	0.1809
Intestinal villus length [μm]	752.95	685.82	789.81	27.74	0.1774	0.1931	1.0
Intestinal villus width [μm]	93.39	111.47	109.16	2.92	0.0001	0.8442	0.0006
Crypt depth [μm]	123.65	120.58	129.95	2.98	1.000	0.1889	0.7826
Collagen – intestinal (%)	19.75	18.57	20.51	0.56	0.3425	0.0609	0.6354
Collagen – liver (%)	8.06	8.84	6.73	0.71	0.5320	0.1222	0.0833

1 CON: Control; H: Hydrogel; HE: Hydrogel + LEO.

2 SEM – standard error of the mean (*n* = 10).

3 Results are means of 5 replicates per treatment.

## Discussion

Modern animal production faces challenges in developing innovative, sustainable, and health-promoting solutions in many agricultural sectors. One promising research direction is the use of alginate hydrogels with immobilized active substances. Due to their unique structure and high water-absorption capacity, hydrogels are excellent carriers for bioactive compounds such as EOs, which are known for their antibacterial, anti-inflammatory, antioxidant, and aromatic properties (Adaszyńska-Skwirzyńska and Szczerbińska, 2019). This combination offers new opportunities for applications in agriculture, the feed industry, and environmental protection. In broiler chicken farming, the initial production stage is crucial, as chicks are exposed to numerous environmental factors from the moment of hatching. Therefore, the first days of life can significantly impact the entire production cycle and its final economic outcome. Chicks differ physiologically from adult birds, with gut microflora and immune system developing during the first few days of life (Hesabi et al., 2022). Early access to feed and water is essential, as feed intake stimulates the development of the gastrointestinal tract and digestive system (Marzok et al., 2025). Delays in feeding or drinking water can hinder the maturation of lymphoid tissue associated with the intestines (*Bursa Fabricii*), while prompt water provision improves nutrient absorption. Administering hydrogels with immobilized active substances during the first days of life allow rapid water replenishment, and their green capsule form also supports natural chick behaviors, reducing stress associated with hatching, transport, and environmental changes (Hesabi et al., 2022). Supplementation with EOs promotes appetite and digestive enzyme secretion (Adaszyńska-Skwirzyńska and Szczerbińska, 2017). Furthermore, hydrogel capsules encourage feeding, supporting adaptation to FI, which is essential for full gastrointestinal peristalsis. In the present *in vivo* experiment, the addition of hydrogel capsules containing immobilized LEO exerted a positive effect on broiler production parameters, particularly on the feed conversion ratio (FCR), which decreased to 1.53 kg/kg compared with 1.58 kg/kg in the control group. The improved feed efficiency may result from the stimulatory effect of the EO on digestive enzyme activity and gut microflora, as also observed in our previous studies (Adaszyńska-Skwirzyńska and Szczerbińska, 2019; Adaszyńska-Skwirzyńska et al., 2021), where LEO was administered as a drinking water supplement. In this study, the improvement in FCR was not associated with a significant increase in body weight during the early rearing period; however, at the final fattening stage, birds in group HE had higher body weights, confirming the positive effect of supplementation on production efficiency. It should be noted that LEO components, including linalool, have anti-inflammatory and antioxidant properties by inhibiting the NF-κB cascade and reducing proinflammatory cytokines (IL-1β, IL-8, etc.), as confirmed in both *in vitro* and *in vivo* models (Pandur et al., 2021). The anti-inflammatory effect may help maintain intestinal barrier integrity, reduce intestinal wall inflammation, and improve nutrient absorption, which can contribute to better feed efficiency and lower energy loss due to inflammatory responses (Pandur et al., 2021; Ding et al., 2022). Analysis of the gut microbiota showed that hydrogel capsules containing immobilized LEO effectively reduced the populations of *E. coli* and coliform bacteria while maintaining stable levels of lactic acid bacteria. This indicated that the supplement exerted selective antimicrobial activity without disrupting the balance of beneficial gut microbiota. It should be emphasized that the *in vivo* experiment was not conducted under conditions of experimental infection or challenge with multidrug-resistant *E. coli*. Therefore, the observed reduction in intestinal *E. coli* and coliform counts reflects modulation of the natural gut microbiota rather than a direct therapeutic effect against pathogenic strains. A similar selective reduction of pathogenic bacteria while preserving probiotic microflora was reported by Zeng et al. (2020) for other EOs used in poultry nutrition. The use of an alginate hydrogel as a carrier helped maintain LEO activity by reducing its volatility and degradation. These results support the use of immobilization technology to improve the stability and bioavailability of plant-derived active compounds (Ahmed, 2015; Huang et al., 2024). Unlike earlier reports by Özlü et al. (2022), who did not observe a beneficial effect of the hydrogel alone on broiler performance, in the present study, capsules containing EO exerted a synergistic effect resulting from the combination of hydrogel properties and the plant-derived bioactive compound. Hydrogel capsules act as a protective barrier, reducing volatility and oxidation, thereby preserving the EO and allowing its gradual release in the intestine. This increases the bioactivity of the oil at the site of action, which may explain the observed reduction in *E. coli* population. According to Huang et al. (2024), alginate encapsulation and modified hydrogel technologies improve the stability and bioavailability of active compounds in applications.

The absence of significant changes in key blood biochemical parameters (ALT, AST, creatinine, uric acid) indicates that both the hydrogel and LEO are safe for birds at the administered dose and do not impair liver or kidney function. This is consistent with our previous research (Adaszyńska-Skwirzyńska and Szczerbińska, 2019), in which we observed no disturbances in biochemical parameters in broilers receiving LEO in drinking water. The safety of hydrogel capsule administration is supported by histological examinations of the intestines and liver, which showed no pathological changes or tissue morphology disturbances. The only significant difference was a greater villus width in birds receiving hydrogel supplementation, which may indicate a positive effect of the additive on the absorptive surface of the small intestine. Increased villus width, with maintained length and crypt depth, may indicate a more developed mucosal structure, which could potentially facilitate nutrient absorption (Ibrahim et al., 2021).

The search for new bioactive compounds is crucial in the context of bacterial drug resistance. The One Health concept is an approach that recognizes human health as closely linked to the health of animals and the shared environment (Deiana et al., 2024). The issue of antibiotic resistance spans interconnected areas, including human and livestock health, as well as antibiotic residues in food and the environment. Poland is the largest producer of poultry meat in the European Union, and pathogenic *E. coli* in birds causes various extra-intestinal infections. Treatment of these infections is increasingly difficult due to the emergence of multidrug-resistant strains, which may pose direct or indirect risks to humans as consumers of poultry products (Shtylla et al., 2023; Deiana et al., 2024; Nawaz et al., 2024). For this reason, alternative methods are being sought to control antibiotic-resistant bacterial infections in poultry using phytobiotics, which can be effective and safe, do not require a withdrawal period, and have a positive effect on animal welfare (Ibrahim et al., 2021; Adaszyńska-Skwirzyńska et al., 2025b). The use of natural alternatives, such as LEO immobilized in hydrogel, for the prevention and support of livestock health may directly contribute to limiting the use of therapeutic antibiotics in agriculture (Adaszyńska-Skwirzyńska et al., 2025a). This reduces selective pressure on bacteria and slows the development of resistance, which is crucial for public health. *E. coli* has long been recognized as a common cause of infections in chicks, leading to significant economic losses (Nawaz et al., 2024). Avian pathogenic *E. coli* (**APEC**) strains are part of the natural gut microbiota of healthy birds. In the presence of predisposing factors such as stress, high stocking density, poor housing conditions, and virus-related immunosuppression, these bacteria can secondarily cause disease symptoms. APEC strains are responsible for respiratory infections, systemic infections, omphalitis, and fibrous-purulent inflammation of the skin and subcutaneous tissue (cellulitis). Most commonly observed are infections of the yolk sac, resulting from pathogen presence on the eggshell (Koutsianos et al., 2020; Shtylla et al., 2023; Nawaz et al., 2024). This results in embryo mortality or bird losses up to the third week of life. The most important diseases associated with APEC strains are respiratory tract and air sac infections, which can progress to bacteremia and may subsequently manifest as generalized serous membrane inflammation (polyserositis) (Koutsianos et al., 2020; Brava et al., 2024). Due to the relatively short 5 to 6-week fattening period, broiler chicks are less frequently exposed to bacterial infections than other livestock species. However, in many cases, antibiotic treatment during rearing is necessary, and its effectiveness is increasingly limited by bacterial drug resistance (Monte et al., 2017; Rouger et al., 2017). After slaughter, poultry carcasses are exposed to cross-contamination much more frequently than meat from other livestock species. Due to the rapid production rate, contamination by bacteria present in the gastrointestinal tract is a commonly occurring phenomenon. Contamination can occur at multiple stages, both during rearing and transport of chicks, as well as in the slaughterhouse during slaughter and post-slaughter processing. Bacteria present in the gastrointestinal tract of birds can reach the surface of the meat during intestinal damage, posing a potential risk to consumer health (Coleman et al., 2003; Rouger et al., 2017). The results have confirmed that LEO shows antibacterial activity against *E. coli* strains, including multidrug-resistant ones. Recent studies indicate that Gram-negative bacteria show greater resistance to essential oils due to the presence of a hydrophilic polysaccharide chain, which forms a barrier against the hydrophobic molecules of EOs (Hou et al., 2022; Khwaza and Aderibigbe, 2025). According to Kalemba and Kunicka (2003), the biological activity of EOs depends on the chemical nature of their main components and decreases in the following order: phenols > aldehydes > ketones > alcohols > esters > ethers > hydrocarbons. The LEO used in the experiment had a high content of monoterpenic alcohols (linalool) and esters (linalyl acetate). The analyses showed that linalool exhibited the strongest antibacterial activity of the LEO components tested, which was consistent with literature data confirming the strong antibacterial properties of linalool (Herman et al., 2016; Guimarães et al., 2019; Parasuraman et al., 2022; Krapež et al., 2024). Other researchers suggest that linalool may affect bacterial cell membrane properties by increasing its permeability, leading to the leakage of cytoplasmic components and, consequently, bacterial cell death (Guimarães et al., 2019; Mączka et al., 2022; Parasurman et al., 2022).

## Conclusion

The LEO under study demonstrated *in vitro* antibacterial activity against clinical *E. coli* strains, and dietary supplementation of broilers with alginate hydrogel containing immobilized LEO positively affected production parameters and intestinal microbiota. The study showed that despite no significant differences in feed intake, the group supplemented with hydrogel capsules containing immobilized LEO achieved a lower FCR, indicating more efficient nutrient utilization. This is particularly relevant given rising feed costs and the need to optimize animal production. These results are significant for the poultry industry, as they provide a natural, environmentally friendly alternative to conventional antibiotic growth promoters, which are now banned. This supports further research on the use of hydrogel technologies for delivering phytobiotics in poultry production. Future studies should investigate different administration periods and inclusion levels of encapsulated LEO, assess antimicrobial resistance gene profiles, and evaluate the efficacy of the preparation under experimental infection with multidrug-resistant *E. coli* strains, which was not feasible because the experiment was conducted under commercial production conditions.

## Ethics approval and consent to participate

The experiment was conducted on a commercial farm (Veterinary Identification Number 32044946) under the supervision of the district veterinarian in Goleniów and the West Pomeranian University of Technology in Szczecin. Approval was obtained from the Local Ethics Committee for Animal Experiments in Poznań (PL12/10/2023). The study adhered to ethical standards, ensuring no pain, suffering, distress, or lasting harm to the animals. Feed and water were provided ad libitum. All procedures involving animals were conducted in full compliance with Act No. 1580/2023 on the protection of animals against cruelty. The manuscript has been in accordance with the ARRIVE 2.0 guidelines.

## Consent for publication

Not applicable.

## Availability of data and material

All relevant data are included in the supplementary material, further inquiries can be directed to the corresponding author.

## CRediT authorship contribution statement

**Michalina Adaszyńska-Skwirzyńska:** Writing – review & editing, Writing – original draft, Validation, Resources, Project administration, Methodology, Investigation, Funding acquisition, Formal analysis, Data curation, Conceptualization. **Sławomir Zych:** Writing – review & editing, Resources, Investigation, Formal analysis. **Marta Grabowska:** Writing – review & editing, Formal analysis, Data curation. **Mateusz Bucław:** Software, Resources, Methodology, Data curation. **Danuta Majewska:** Resources, Methodology, Investigation, Formal analysis. **Danuta Szczerbińska:** Visualization, Supervision, Methodology, Formal analysis, Data curation. **Adam Lepczyński:** Supervision, Resources, Project administration, Methodology, Investigation, Formal analysis, Data curation, Conceptualization. **Paweł Konieczka:** Visualization, Validation, Supervision, Resources, Project administration, Methodology, Conceptualization.

## Disclosures

The authors declare that they have no known competing financial interests or personal relationships that could have appeared to influence the work reported in this paper.
